# Metal electrode integration on macroporous silicon: pore distribution and morphology

**DOI:** 10.1186/1556-276X-7-395

**Published:** 2012-07-16

**Authors:** Gilles Scheen, Margherita Bassu, Laurent A Francis

**Affiliations:** 1 Institute of Information and Communication Technologies, Electronics and Applied Mathematics, Université Catholique de Louvain, Place du Levant, 3, Louvain-la-Neuve, B-1348, Belgium

**Keywords:** Macroporous silicon, Integration, Metal electrodes, Electrolyte composition, Pattern

## Abstract

In this work, a new approach for the one-step integration of interdigitated electrodes on macroporous silicon substrates is presented. Titanium/gold interdigitated electrodes are used to pattern p-type silicon substrates prior the anodization in an organic electrolyte. The electrolyte characteristics, conductivity, and pH have been found to affect the adherence of the metal layer on the silicon surface during the electrochemical etching. The impact of the metal pattern on size distribution and morphology of the resulting macroporous silicon layer is analyzed. A formation mechanism supported by finite element simulation is proposed.

## Background

Silicon anodization in hydrofluoric acid (HF)-based organic electrolyte is a simple and effective technique to obtain macroporous silicon (MPS) on p-type substrates. Macroporous silicon has been demonstrated as an interesting material for a number of applications such as silicon micromachining [1,2], photonic [3], and sensing [4,5]. The integration of metal electrodes on macroporous silicon surface is of paramount importance in case of sensing applications [6]. In silicon microtechnologies, metal electrodes are usually obtained by patterning metal layers evaporated or deposited on the substrate surface. Due to the high macroporous silicon surface roughness, the patterning of electrodes with dimensions below 5 *μ*m is a challenge. An alternative approach is to pattern the metal electrodes on the silicon surface prior the electrochemical etching. In this case, the presence of the metal on the surface affects pore distribution and morphology. In this work, the macroporous silicon characteristics are investigated as a function of the geometry (width and pitch) of metal pattern and modeled using finite element simulations.

## Methods

Metal interdigitated electrodes were patterned on the surface of p-type silicon substrates (10 to 20 *Ω*·cm, (100) orientation) using standard lift-off. The electrodes consist of 1400-*μ*m long Au ribbons (100-nm thick). Au was chosen because it is HF resistant. A 5-nm thick Ti layer was used to promote the Au surface adherence. Ti was chosen instead of Cr because the combination of Cr and Au forms an electrochemical local element, giving rise to the metal layer detachment after few minutes of electrochemical etching [7]. The metal was evaporated all over the wafer using a dual e-beam evaporator (e-gun Balzers, Liechtenstein). The nondesired metal layers on the wafer were removed by dissolving the prepatterned underlying resist in acetone. Three different geometries (widths and pitches, WP (*μ*m)) were tested on the same sample: WP1 = 2-2, WP2 = 5-2, and WP3 = 2-5. The prepared samples were then electrochemically etched to form the macroporous silicon layer. All the experiments were carried out at room temperature, and the solution was stirred to avoid inhomogeneities due to the gas accumulation on the surface. A platinum grid was used as the cathode, while the anode was the silicon substrate. A 200-nm thick aluminum layer was evaporated on the wafer back side, and an electrical contact was made by placing an aluminum plate in contact with it. A mixture of HF and N,N-dimethylformamide (DMF) (HF (48%):DMF = 1:4) was used as electrolyte. DMF is not stable in presence of acids like HF and is, thus, partly hydrolyzed back into formic acid and dimethylamine [8]. The reaction is exothermic and gives rise to the increase of the solution temperature. Solution pH and conductivity were measured after the cooling of the solution at room temperature. A pH of 4 and a conductivity of 2 mS/m were measured. The hydrolysis of the DMF is not limited to the preparation moment. As a consequence, the properties of the solution were observed to change to reach a threshold value. For a 20-day-old solution stored in a closed bottle, it was measured with a pH of 6 and a conductivity of 30 mS/m, indicating that a bigger fraction of the DMF was hydrolyzed. Both as-prepared and 20-day-old solutions were used as electrolytes.

The enhanced conductivity of the old solution allows to use higher current densities for lower applied voltage. In both cases, the macroporous silicon layers were formed, keeping constant the etching current density *J*_etch_ at a value lower than the critical current density *J*_ps_=182 mA/cm^2^ at which the silicon surface electropolishing is produced [9].

## Results and discussion

**Figure 1:**
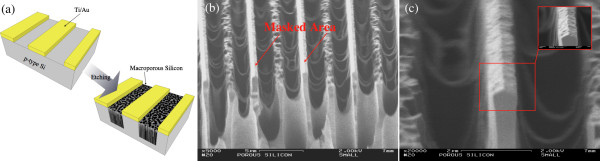
**Schematic representation of the process.** (**a**) SEM cross-sectional tilted view of a macroporous layer grown on a Ti/Au prepatterned substrate (WP1 geometry) (**b**) and (**c**). The etching current was set at the constant value of 91 mA/cm^2^ for 25 min. A fresh HF/DMF mixture was used as electrolyte. The metal layers were released after few minutes of etching.

When the silicon substrate is prepatterned with an HF-resistant material as the Au mask, the pore formation is expected to be limited to the uncovered areas, as sketched in Figure 1a. Figure 1b,c shows the SEM cross-sectional tilted view of a macroporous silicon layer resulting from the etching of a Ti/Au prepatterned surface (WP1 geometry). An as-prepared HF/DMF mixture was used as electrolyte (pH = 4, conductivity = 2 mS/m). The etching current density was set at the constant value 91 mA/cm^2^ for 25 min. An etching voltage (*V*_etch_)≈6*V* was necessary to sustain such current. A slow increment of *V*_etch_as a function of the etching time was observed in all experiments. The metal mask was detached by the substrate before the end of the etching. As shown in Figure 1c, the underetching of the masked area reduced the contact area between the metal layer and the silicon surface up to the detachment of the masking layer. The observed etching profile induces to suppose a catalytic role of the Ti/Au layer in the etching of the silicon in the close proximity of the metal layer, similar to what happened in the electroless metal-assisted chemical etching of silicon [10].

**Figure 2:**
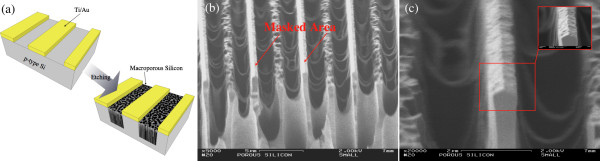
**SEM cross-sectional tilted view of a macroporous layer grown on Ti/Au patterned silicon substrates.** Geometry WP1 (**a** and **b**) and geometry WP2 (**c**). A 20-day-old HF/DMF mixture was used as electrolyte. The current density was set at the constant value of 91 mA/cm^2^ for 25 min.

Figure 2 shows the SEM cross-sectional tilted view of Ti/Au prepatterned samples (geometry WP1 (a) and (b) and geometry WP2 (c)) etched used as an electrolyte of a 20-day-old HF/DMF mixture. As in the previous case the etching current density was set at the constant value 91 mA/cm2 for 25 min.

The different characteristics of the electrolyte (pH =6, conductivity = 30 mS/m) affected the etching parameters as well as the morphology of the resulting macroporous layer. A lower etching voltage, *V*_etch_≈1.5 V, was necessary to sustain *J*_etch_ = 91 mA/cm^2^. Even in this case, a slow increase of the etching voltage as a function of the etching time was observed. A different etching profile was observed in close proximity of the masking layer. The metal layer was sufficiently adherent to the silicon substrate to sustain the etching process and the sample cutting for analysis.

**Figure 3:**
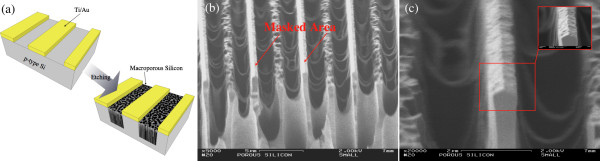
**Comparison of experimental and simulation results for the WP1 geometry.** Top (**a**) and cross-sectional tilted views (**b**) of the macroporous layer. The current density was set at the constant value of 26 mA/cm^2^. The frame (**c**) shows the COMSOL-simulated cross-section. The shading represents the holes current density; the streamlines, the hole flux streamlines.

Using as-prepared solutions as well as 20-day-old solutions, the pore distribution was affected by the presence of the Ti/Au mask. More specifically, the pores tend to grow in proximity of the metal edges. As a consequence, the pores grow with a random distribution in the direction parallel to the metal strips, and they grow with a regular spacing in the transverse direction. In the case of geometries with 2-*μ*m pitches, a silicon wall in the middle of the unmasked area is formed. The depth and the high roughness of the top surface of this wall are due to the nucleation phase in which the initiation of the pore growth follows an almost-uniform etching of the surface. The dimension of this wall, as well as the pore diameter, depends on the applied current density. Figure 3 shows the top and cross-sectional SEM views of a MPS layer with metal electrodes of WP1 geometry. A 20-day-old HF/DMF mixture was used as electrolyte. The etching current density was set at the constant value of 26 mA/cm^2^ for 25 min. Both walls, the one sustaining the metal layer and the one between the electrodes, are larger with respect to the samples shown in Figures 1 and 2 because of the smaller etching current. On the contrary, the thinner perpendicular wall separating the pores does not show a strong dependence on the applied etching current.

**Figure 4:**
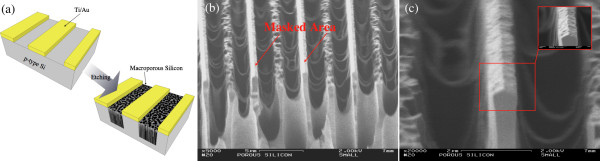
**Comparison of experimental and simulation results for the WP3 geometry.** SEM cross-sectional views of the macroporous layer (**a** and **b**). COMSOL-simulated cross-section (**c**)

Two-dimensional simulations of the interface silicon/mask/electrolyte were performed using COMSOL MultiphysicsⓇ (COMSOL AB, Stockholm, Sweden) to better understand the etching behavior of how the metal pattern geometry affects the macroporous layer morphology. To model the electrolyte/mask/silicon interface behavior, we considered a semiconductor/metal contact at the front side of a p-type wafer (10^15^ at./cm^3^) with a bias of -1 V applied on the conductor border at 30 *μ*m from the semiconductor surface. Figure 3c shows the simulated cross-section of a metal pattern with geometry WP1. Holes current density (shading) and hole flux streamlines are shown. The flux streamlines follow the electric field lines. The simulation results show that, in the initial stage of the etching, the current density is higher at the metal electrode edges and converges with the field lines towards the metal strip corners. A direct consequence of the current density distribution is the experimentally observed growth of the pores in proximity of the mask edges. Even if during the nucleation phase pores start to grow in all the unmasked area, the pores on the corners of the mask are privileged by the higher current density that leads to a bigger growth rate. In case of smaller pitches, this behavior is reflected in the formation of the middle walls because the space between the metal strips is not sufficient to support the formation of another pore. In case of bigger spacing between the metal strips, the formation of the thicker middle wall is avoided. In fact, as clearly visible in Figure 4 that shows the SEM cross-sectional tilted view (a and b) and the simulated cross-section for a Ti/Au prepatterned surface with geometry WP3, the pores on the middle go further the nucleation phase. The higher current at the mask edges in the initial stage of the etching is reflected in a bigger nucleation and pore depth.

## Conclusions

A new approach for the integration of metal electrodes on macroporous silicon was shown. The impact of etching parameters as electrolyte characteristics and etching current on the formation of silicon macropores on Ti/Au prepatterned substrate was analyzed. It was shown that the electrolyte consisting of an as-prepared HF/DMF mixture induces catalytic phenomenon at the silicon-metal interface and the consequent metal underetching and detachment. On the other hand, electrolytes consisting in 20-day-old HF/DMF mixtures, in which the DMF hydrolization gives rise to increased conductivity and pH, allow the integration of metal interdigitated electrodes on macroporous silicon. In both cases, the pore distribution and morphology are affected by the presence of the metal pattern. Pores grow faster in close proximity of the metal strip edges, leading to the formation of walls in the middle of the uncovered area for small pitches. The experimental results were in agreement with the COMSOL simulation results that showed a current density distribution influenced by the enhanced electric field at the metal edges.

Future work will be devoted to study the influence of the electrolyte composition, surface state, and metallic materials on the catalytic behavior of the metal layer, with the important consequence on the adhesion of the metal layer at the silicon surface.

## Competing interests

The authors declare that they have no competing interests.

## Authors’ contributions

GS and MB conceived of the experiments and drafted the manuscript. GS conceived of the COMSOL simulations. LAF supervised the research activity. All authors read and approved the final manuscript.

## Authors’ information

GS is a PhD student of the Université Catholique de Louvain (UCL). MB is post doctorate research assistant at the UCL. LAF is an associate professor at the UCL. All authors are members of the group Sensors, Microsystems and Actuators Laboratory of Louvain (SMALL) chaired by LAF.

## References

[B1] Barillaro G Bruschi P Diligenti A Nannini A Fabrication of regular silicon microstructures by photoelectrochemical etching of silicon Phys Status Solidi C 2005 2 9 3198 3202 10.1002/pssc.200461110

[B2] Bassu M Surdo S Strambini L Barillaro G Electrochemical micromachining as enabling technology for advanced silicon microstructuring Adv Funct Mater 2012 22 1222 1228 10.1002/adfm.201102124

[B3] Grüninga U Lehmann V Ottow S Busch K Macroporous silicon with a complete two-dimensional photonic band gap centered at 5 μm Appl Phys Lett 1996 68 6 747 749 10.1063/1.116729

[B4] Betty CA Lal R Sharma DK Yakhmi JV Mittal JP Macroporous silicon based capacitive affinity sensor - fabrication and electrochemical studies Sens Actuators, B 2004 97 334 343 10.1016/j.snb.2003.09.008

[B5] Ozdemira S Gole JL A phosphine detection matrix using nanostructure modified porous silicon gas sensors Sens Actuators, B 2010 151 274 280 10.1016/j.snb.2010.08.016

[B6] Wang Y Parka S Yeow JT Langner A Müller F A capacitive humidity sensor based on ordered macroporous silicon with thin film surface coating Sens Actuators, B 2010 149 136 142 10.1016/j.snb.2010.06.010

[B7] Lammel G Schweizer S Schiesser S Renaud P Tunable optical filter of porous silicon as key component for a MEMS spectrometer J Microeletromech Sys 2002 11 6 815 827 10.1109/JMEMS.2002.803278

[B8] Eberling CL Kirk RE, Othmer DF, Grayson M, Eckroth DV Dimethylformamide Kirk-Othmer Encyclopedia of Chemical Technology. Volume 11 1980 John Wiley & Sons, New York 263 268

[B9] Lehmann V Electrochemistry of Silicon - Instrumentation Science Materials and Applications 2002 Wiley-VCH Verlag, Berlin

[B10] Huang Z Geyer N Werner P de Boor J Gösele U Metal-assisted chemical etching of silicon: a review Adv Mater 2011 23 285 308 10.1002/adma.201001784 20859941

